# Supplementary material for: Left ventricular systolic function impairment in children after balloon valvuloplasty for congenital aortic stenosis assessed by 2D speckle tracking echocardiography
Source: PLoS One. 2021 Apr 29;16(4):e0248862. doi: 10.1371/journal.pone.0248862 (PMC8084170; doi:10.1371/journal.pone.0248862)
Supplement: Attachment — Submitted filename: Responses to Reviewers.doc [file pone.0248862.s002.doc]

We are very grateful to both reviewers for taking their time to review the manuscript and share their valuable comments. Please find below our replies to the Reviewers comments together with adjustment that were made to manuscript accordingly.

We believe our revised manuscript represents a significant improvement over our initial submission

**Reviewer #1**

1. Abstract – long term – this needs to be clearly defined in months with a range/sd; see comment above

The information in the abstract was completed,as suggested –„ more than 36 months, 115.6±57.8 months”. The information concerning the observation period is also given in line 71 of the chapter "Methods" – „ Only patients who had undergone BAV more than 3 years before were included, with the mean duration of the post-BAV period being 115.6±57.8 months (range: 37 to 216 months)”.

2. Abstract – the GLS should be just one decimal point (-19.69 should be -19.7); I don’t think strain is accurate to 4 significant figures.

The changes have been done –„GLS (-19.69±2.22% vs. -22.31±1.5%....”has been changed to „GLS (-19.7±2.22% vs. -22.3±1.5%...”.

3. Abstract – what other variables besides GLS were in the multivariable model; this should be included in the abstract too

The information was added to the abstract as suggested - Multivariable logistic regression analysis included: instantaneous peak systolic Doppler gradient across aortic valve (PG_max_), grade of aortic regurgitation (AR), left ventricular mass index (LVMI), left ventricular relative wall thickness (LVRWT), left ventricular end-diastolic diameter (LVEDd), peak systolic mitral annular velocity of the septal and lateral corner (S’spt, S’lat), LVEF before BAV and time after BAV and showed that the only predictor of reduced GLS was LV eccentric hypertrophy [odds ratio 6.9; (95% CI: 1.37–12.5), *P*=0.045].

4. Abstract – the authors describe “left ventricular systolic dysfunction” in the study patients – I would argue that the strain may be within the normal limits. I would state lower strain values than the control patients in the study; please see my prior comment above

We corrected the sentence as suggested: „Regional (basal, middle and apical segments) strain and strain rate were also lower compared with control group”.

We corrected the term dysfunction to impairment.

5. Abstract – balloon is mispelled

the word „balloon”in abstract has been written correctly.

6.Introduction – The authors state: “The conventional two-dimensional echocardiography (left ventricular ejection fraction [LVEF] assessment) fails to reveal LV systolic function impairment for a long duration of the disease.” Do we know for sure that there is systolic function impairment? I would argue that the conventional measurements show presumed normal function or even normal FS or EF.

The following 2 sentences – „The conventional two-dimensional echocardiography (left ventricular ejection fraction [LVEF] assessment) fails to reveal LV systolic function impairment for a long duration of the disease. Studies conducted in adults with AS suggest that LV systolic function impairment, once discovered by conventional methods (LVEF), already indicates an advanced degree of LV dysfunction [4]”

have been replaced by a sentence - „The study by Capoulade et al.[4] presenting the relationship between a reduced ejection fraction and higher mortality in adult patients with aortic valve stenosis, indicates that decreased EF is a marker of advanced stage of the disease”.

This sentence reflects more precisely what the authors wanted to express.

7. Introduction – The authors state that the diminished LVEF in adults “already indicates an advanced degree of LV dysfunction.” How do we know this? What is a marker of this?

If the reduction of EF is associated with higher mortality, it can be indirectly concluded that it is associated with the severity of the disease.

8. Methods – how do we know that the control group is healthy? Were these volunteers? Were these patients that happened to come for indications that were benign and discovered to have innocuous disease? Why are there not an equal number of control patients? Did the controls not get matched to the age/size of the study patients?

The control group consisted of healthy volunteers. Volunteers, who were found to have any kind of pathology of circulatory system or other systems, based on an interview and an echocardiographic examination, were excluded from the study.Volunteers, who were taking any kind of drugs were also excluded from the study.

The control group consisted of 62 volunteers matched to the age, gender, body weight, body surface area and heart rate of the study group.

We’d like to thank the Reviewer for highlighting the issue of similarity of patients and controls according to key factors: age and gender. We focused on ensuring homogeneity of the patient and control groups. That’s why we noticed no differences in age and gender between both groups (p>0,6). Since our aim was to determine the cut-off point for GLS values with as little error as possible we decided to include more healthy subjects into the controls. Our decision to abandon the equal-study-groups-strategy resulted in not being able to pair patients according to a 1:1 scheme, however we believe it adds value to the paper.

9. Table 1 – The authors have a 0,7 when it should be a decimal place. 0.7 should have 2 significant figures in the p-value section like the other p-values. Other than IVSd, LVPWd, LVRWT, the other values should have only one decimal place.

The suggested changes in table 1 were made. The number 0,7 was changed to 0.7. P-value 0.7 was changed to 0.70. Apart from IVSd, LVPWd, LVRWT, the other values in columns 2 and 3 have been changed to one decimal place. The changes are marked in yellow.

10. Methods – LVRWT<0,44 should be 0.44 and did the authors use a mass/volume ratio to detect eccentric hypertrophy? Is there a reference for LVRWT the authors can supply to justify the eccentric hypertrophy definition?

The number <0,44 was changed to <0.44.

We didn’t use a mass/volume ratio to detect eccentric hypertrophy, but used LVMI and LVRWT. The ratio of LV mass to end-diastolic volume (M/V) is closely related to the LVRWT. Changes in this ratio (mass/volume) should carry the same implications as changes in LVRWT.

The relationship of M/V and LVRWT is shown in a diagram below.

(Gaasch, W. H., &Zile, M. R., Left Ventricular Structural Remodeling in Health and Disease, 2011, JACC, 58, 1733–1740.)

The LVEH is defined as an increase in LVMI with normal of LVRWT. Although the exact cut-points of LVRWT and LVMI as well have varied. In many studies a cut-point value for LVRWT is 0.44. [references 1-8].

In this study, we used the cut-off point (as the upper limit of normal) of the 95th percentile for LVMI [reference 2].

References for LVEH:

1. De Simone G, Left Ventricular Geometry and Hypotension in End-Stage Renal Disease: A Mechanical Perspective, J Am Soc Nephrol, 2003, 14: 2421–2427,
2. Daniels SR, Kimball TR, Morrison JA, Khoury P, Meyer RA. Indexing left ventricular mass to account for differences in body size in children and adolescents without cardiovascular disease. Am J Cardiol, 1995; 76: 699–701.
3. Dávila, D. F., Donis, J. H., Odreman, R., Gonzalez, M., Landaeta, A. Patterns of left ventricular hypertrophy in essential hypertension: Should echocardiography guide the pharmacological treatment? 2008, International Journal of Cardiology, 124(2), 134–138.
4. Kosmala W, Przewłocka-Kosmala M, Orzeszko J, Karolko B, TNF-α and Left Ventricular Geometry in Patients with Essential Hypertension, Adv Clin Exp Med 2005, 14, 4, 677–682
5. Muiesan ML, Salvetti M, Monteduro C et al, Left Ventricular Concentric Geometry During Treatment Adversely Affects Cardiovascular Prognosis in Hypertensive Patients, Hypertension. 2004; 43:731-738.
6. Koren MJ, Devereux RB, Casale PN, Savage DD, Laragh JH. Relation of left ventricular mass and geometry to morbidity and mortality in uncomplicated essential hypertension. Ann lntern Med. 1991;114:345–352. )
7. McBride SM, Flynn FW, Ren J, Cardiovascular Alteration and Treatment of Hypertension, Endocrine, 2005, *28, 199–207.*
8. Lavie, C. J., Patel, D. A., Milani, R. V., Ventura, H. O., Shah, S., Gilliland, Y. Impact of Echocardiographic Left Ventricular Geometry on Clinical Prognosis. Progress in Cardiovascular Diseases,2014, 57(1), 3–9.

11.Methods – The authors state “By using continuous-wave Doppler (CW-Doppler) and color Doppler an assessment of the LV-Ao instantaneous peak systolic Doppler gradient (PGmax) and the grade AR (on a 4-point grading scale) were made.” I believe an assessment “was” made not “were” made.

The sentence „By using continuous-wave Doppler (CW-Doppler) and color Doppler an assessment of the LV-Ao instantaneous peak systolic Doppler gradient (PG_max_) and the grade AR (on a 4-point grading scale) were made” has been changed to „By using continuous-wave Doppler (CW-Doppler) and color Doppler an assessment of the LV-Ao instantaneous peak systolic Doppler gradient (PG_max_) and the grade AR (on a 4-point grading scale) was made”.

12. Methods – pulse-wave should be pulsed-wave Doppler (line 95)

The term „pulse-wave Doppler” has been corrected „pulsed-wave Doppler” (line 102)

13. Methods – line 107, did the authors or the software reject poorly tracking segments?

The sentence „After the manually traced of the endocardium border and the width of the region of interest the software automatically tracked and accepted segments of good tracking quality, rejecting poorly tracked segments” was changed to „After the manually traced of the endocardium border and the width of the region of interest the software automatically tracked and accepted segments of good tracking quality, automatically rejecting poorly tracked segments”.

14. Methods – line 115-116, the authors used the control group to establish cut-off values with a standard deviation. The authors should look at established normal values for strain for the software utilized in this study. If none exist, then the authors should state this.

Yet, speckle tracking standards are available in healthy children, and STE anomalies have been reported in many situations involving myocardial dysfunction in children. Nevertheless, unlike in the adult population, the feasibility of STE analysis in pediatrics has not been clearly studied, from birth to teenage. There are no established normal ranges of left ventricular strain using 2D STE for the paediatric population utilizing commercially available QLab software (Philips), that can be considered as a mandatory standard. This information has been added to the manuscript (methods section, line 123).

15. Methods – Statistical Analysis – ICC and interobserver variability is not mentioned here when these comparisons are in the results. How was percentage difference calculated? This makes a significant difference. Did the authors compare percentage difference (100%*(observer1-observer2)/(average of observers) or just the absolute difference such as -17.1% vs -16.1% (1% difference). The first yields a 6% difference while the second is just a 1% difference. I highly recommend that it should be a percentage difference (the first one with 6%) since that clearly tells the variability.

To clarify the variability calculation method, the following sentence „Intraobserver variability was assessed using intraclass correlation and absolute difference divided by the mean of the repeated observations expressed as percentage” has been changed to the sentence „Intra- and interobserver variability were assessed using intraclass correlation and absolute difference between the investigators' measurements divided by the average value of both measurements expressed as percentage”.

Intra- and interobserver differences was assessed according to the formula given below.

Three sentences included in the results section - „For intra- and interobserver variability, echocardiographic measurements in 12 randomly selected patients were repeated. The measurements were performed on the same cardiac cycle. Intra- and interobserver variability were assessed using intraclass correlation and absolute difference between the investigators' measurements divided by the average value of both measurements expressed as percentage” have been moved to the methods section.

16. Results – The follow-up data after BAV should be given in line 133 after the description of BAV. This way the reader knows precisely how long there has been remodeling.

The sentence concerning the long-term follow-up after BAV - „ Only patients who had undergone BAV more than 3 years before were included, with the mean duration of the post-BAV period being 107.5±57.8 months (range: 37 to 216 months)” has been moved to the methods section (lines 71) as suggested.

17. Results – The AR and AS patients overlap and a VENN diagram or something equivalent to show – pure AS, pure AI and mixed disease would be much more helpful

The following diagram was inserted to the paper instead of descriptive information regarding the occurrence of PG_max_ and AS included in results chapter, as suggested.

Figure 1. Characteristic of the study group in regards to the occurrence of PG_max_ and AS.

Abbreviations – see in Table 2.

18. Table 2 – the values should have only one decimal point for the LVEH and no LVEH columns. P-value columns are fine except for the LVEF P-value should be two decimals not one.

Table 2 has been modified according to the suggestions: columns 1 and 2 are given 1 decimal place and column 3 is given 2 decimal places.

19.Results – line 163 what does value greater mean. -19% is technically less than -18% but we know for strain that is not the case. I would state the “The magnitude value” or “The absolute value”. The authors should use just 2 decimal places for strain rate and one for strain on line 163 (ie., -19.31 should be -19.3 while -0.8/s should be -0.80/s).

In the methods section (in line 127), there is an information explaining what the greater value for strain means -„The less negative GLS, GCS, GLSr and GCSr values means the lower systolic function”.

The values of GLS, GCS, GLSr and GCSr have been corrected as suggested.

20. Results – line 166 I would replace the word “impaired” with “less”.

The sentence has been changed „We found LV systolic function to be less in the group of post-BAV patients compared with the control group” as suggested.

21. Results – I strongly urge a subanalysis of GLS for mostly volume loaded left ventricles, mostly pressure loaded left ventricles, and mixed disease to determine if there is a category that is more strongly lower than the normal cut-off values.

The review posed the question of the relationship between the type of predominant valvular defect (stenosis/regurgitation/mixed nature) and the change in GLS. Many thanks to the Reviewer for this question. During data analysis, we’d compared GLS among patients in three subgroups according to dominant valvular defect –we’d found no differences between groups in the Kruskal-Wallis ANOVA test (p=0.84). Due to the number of analyses performed, we had not include this information in the manuscript. After the Reviewer's remark, the information about the analysis performed and its result was introduced into the text of the publication (line 214)

22. Table 3 – p-value 0.9 should be two decimal places. The other values for strain should have one decimal place while strain rate two decimal places.

Table 3 has been changed as suggested.

23. Results – line 177: GLS in the study group correlated positively with LVEH. This makes no sense since higher LVEH made the GLS lower. Are the authors suggesting that the strain improves with more aortic stenosis or insufficiency?

From a mathematical point of view a positive correlation means that the value of one feature increases with the value of the other. In our study, the term positive correlation means that the presence of LVEH results in higher values of GLS, that is, less negative GLS values=lower GLS. In the context of earlier explanation of what lower GLS means, the information concerning positive correlation should be understandable for the reader. We added a diagram - The correlation of GLS with the existence of LVEH (line 197).

Figure 2. The correlation of GLS with the existence of LVEH.

GLS, global longitudinal strain; LVEH, left ventricular eccentric hypertrophy.

24. Table 4/5 – please see prior comments about significant figures/decimal places. The authors should use the abbreviation for LVEH or take out the abbreviation altogether.

Table 4 and 5 has been changed as suggested.

25. Results – lines 227-245 – these paragraphs reiterate what is in the table and is redundant; this needs to be summarized into just the most pertinent findings or the reader will get distracted

The text contained in lines 258-278 was replaced by the following „In the subgroup of patients with LVEH, GLSr and strain rate in the basal, middle and apical segments were significantly lower compared with the non-LVEH subgroup (Table 5). All the patients with reduced GLSr were found to have LVEH. The values of PG_max_ and LV RWT were significantly higher in patients with reduced GLSr compared to the subgroup with normal GLSr (*P*<0.001 and *P*=0.048, respectively) (Table 4).

None of the patients in the study group had a GCSr reduction. GCSr and strain rate in the basal, middle and apical segments did not differ significantly between the study group and the control group (Table 3) and between the subgroup of patients with LVEH and the non-LVEH subgroup. No significant correlations were observed between GCSr and the factors listed in the table 4”.

26. Results – were the interobserver variability results blinded. Were the %differences absolute percent difference or just the difference in strain? Please see my prior comment on reproducibility. The authors report reproducibility on strain. What about strain rate?

Interobserver variability results were blinded. The difference between observers was calculated according to the following formula:

Other explanations are given in point 15.

The variability was calculated for both strain and strain rate. The following sentence „Intraobserver variability was assessed using intraclass correlation and absolute difference divided by the mean of the repeated observations expressed as percentage” has been changed to the sentence „Intra- and interobserver variability was assessed using intraclass correlation and absolute difference between the investigators' measurements divided by the average value of both measurementsexpressed as percentage”. (as given in point 15).

27.Discussion – what disease aortic stenosis/regurgitation/mixed disease causes the biggest difference in strain values from the control group.

We found no differences between the type of predominant valvular defect (stenosis/regurgitation/mixed nature) and the change in GLS (p=0.84, Kruskal-Wallis ANOVA test ).

28. Discussion – line 340 - Notably circumferential strain was no different in the quoted study, similar to this current study.

The following sentence was added to the discussion (line 382) „In addition, circumferential strain was no different in the quoted study, similar to this current study”.

**Reviewer #2**:

1. Abstract: LVEF should be spelled out in the abstract (other abbreviations are included in the abstract)

The term ”LVEF” has been corrected .

2. Materials and Methods
The length and content of the methods section is appropriate, however one minor point is, if the authors could not test inter and intra -observer variability, could they provide a reference where it has been tested in the past? It would make the manuscript stronger.

Intra- and interobserver variability was performed in this study (methods section)
